# Substrate-dependent electronic structure and film formation of MAPbI_3_ perovskites

**DOI:** 10.1038/srep40267

**Published:** 2017-01-13

**Authors:** Selina Olthof, Klaus Meerholz

**Affiliations:** 1 Department of Chemistry, University of Cologne, Luxemburger Straße 116, 50939 Cologne Germany

## Abstract

We present investigations on the interface formation between the hybrid perovskite MAPbI_3_ and various substrates, covering a wide range of work functions. The perovskite films are incrementally evaporated *in situ* while the electronic structure is evaluated using photoelectron spectroscopy. Our results show that there is an induction period in the growth of the perovskite during which volatile compounds are formed, catalyzed by the substrate. The duration of the induction period depends strongly on the nature of the substrate material, and it can take up to 20–30 nm of formal precursor deposition before the surface is passivated and the perovskite film starts forming. The stoichiometry of the 2–3 nm thin passivation layer deviates from the expected perovskite stoichiometry, being rich in decomposition products of the organic cation. During the regular growth of the perovskite, our measurements show a deviation from the commonly assumed flat band condition, i.e., dipole formation and band bending dominate the interface. Overall, the nature of the substrate not only changes the energetic alignment of the perovskite, it can introduce gap states and influence the film formation and morphology. The possible impact on device performance is discussed.

Organo-metal halide perovskites have established their place in the field of solar cell research as one of the most exciting materials currently under investigation. The impressive increase in device efficiency, which occurred during the last years, has led to a surge in interest. For various combinations of organic cations (e.g methylammonium MA, formamidinium FA), metals (Pb, Sn), and halides (I, Br, Cl) the thin film properties have been extensively investigated, and it became clear that the excellent performance is due to the high mobility^1^, high absorption^2^, low exciton binding energy^3^, as well as low trap density^4^. Current research focusses on further improving the morphology^5^,^6^,^7^ and (phase-)stability^8^,^9^,^10^, reducing the current-voltage hysteresis^11^,^12^,^13^, and replacing the lead by environmentally acceptable metals^14^,^15^,^16^,^17^ in order to path the way for the commercialization of devices.

In all this success, is it often overlooked that rather little is understood about the operating mechanisms of the solar cell devices as a whole. A crucial role for achieving high open-circuit voltages and short-circuit currents are optimally aligned electronic levels throughout the device. Due to lack of better knowledge, non-optimized interfaces have been employed, leading to less than optimal device performances. Furthermore, vacuum level alignment and flat-band conditions are commonly assumed when presenting energy level diagrams (see e.g. refs 18,19), neglecting the possible effects of interface dipole formation or band bending which can considerably alter the device performance.

A powerful tool to directly investigate the energetic alignment at these interfaces is UV photoelectron spectroscopy (UPS), which probes the occupied density of states (DOS). The method not only yields the ionization energy (IE), work function (Wf), and injection barrier of films, but due to the high surface sensitivity possible interface dipole formation and band bending can be probed by incrementally building up an interface. So far, several publications have shown studies of device relevant interfaces between the most commonly employed perovskite MAPbI_3_ and an incrementally deposited top layer, of e.g. 2,2′,7,7′-Tetrakis-(N,N-di-4-methoxyphenylamino)-9,9′-spirobifluorene (spiro-MeOTAD)^20^, fullerene C_60_^21^,^22^,^23^, various hole transport layers^24^,^25^, [6,6]-phenyl-C61-butyric acid methyl ester (PCBM)^21^, Au^23^,^26^, or MoO_3_^27^; these studies found various degrees of band bending and/or interface dipole formation. Other research looked at the sequential evaporation of precursor materials, e.g. methylammonium iodide (MAI) on top of PbI_2_^28^ or lead halides on top of MAI^29^. Regarding the influence of substrate work function on the position of the perovskite conduction band (CB) and valence band (VB), a pinning of the Fermi energy at the CB is commonly observed for n-type substrates like TiO_2_, ZnO, and Al_2_O_3_, with a Wf around 4 eV^22^,^30^,^31^. On the other hand, on p-type substrates like poly(3,4-ethylenedioxythiophene)-poly(styrenesulfonate) (PEDOT:PSS), NiO_x_, p-Si, and Cu_2_O the Fermi level moves roughly to mid-gap, showing a Wf around 4.7^22^,^23^,^26^,^30^,^32^. This means that the Fermi level position in the bandgap can be partially tuned by the contacting material, indicating a low intrinsic charge carrier density.

The above mentioned studies have all been done on top of thick perovskite films. Here, the energetic alignment at the buried substrate interface, i.e. the interface dipole and band bending within the perovskite, cannot be accessed and therefore this remains a mostly unexplored topic. To shed light on this interface, incremental thermal evaporation of the perovskite film is necessary which was so far only been done in two recent publications. Zhou *et al*.^33^ investigated the alignment between a ZnO(0001) single crystal and a stepwise evaporated MAPbI_3_ layer. Here, the authors found the formation of a 0.5 nm thick PbI_2_ interface layer that introduced a significant interface dipole and only a negligible band bending in the perovskite layer. In good agreement, Xu *et al*.^34^ found on various substrates an interface related PbI_2_ signature using x-ray diffraction on MAPbI_3_ deposited on top of indium tin oxide (ITO), PEDOT:PSS, Si, and glass. They concluded that at the interface MAI does not efficiently stick to the surface leading to this excess PbI_2_ layer which introduces a barrier and hinders charge transport.

These deviations in the chemical deposition of perovskite films as well as the appearance of transport barriers are important topics that have to be considered for device fabrication. Therefore, careful investigations on a wide range of substrates would help to enhance our fundamental understanding of these processes and could enable further optimization of interfaces which will provide guidance for future stack design and device optimization. Thus, the present work focusses on the investigation of such interfaces by incrementally evaporating the perovskite MAPbI_3_ on four different substrate materials, using the precursor materials MAI and PbI_2_. The substrates are chosen to cover metal oxides and organic materials, as well as a wide range of work functions. They are commonly used in the field of organic electronics and include ITO, molybdenum oxide (MoO_3_), PEDOT:PSS, and polyethylenimine ethoxylate (PEIE). We use UV photoelectron spectroscopy (UPS) to look at changes in Wf, DOS, and valence band onset as well as x-ray photoelectron spectroscopy (XPS) to study film formation, film stoichiometry, and chemical shifts during layer growth.

## Results and Discussion

*In-situ* UPS and XPS measurements were performed on MAPbI_3_, which was stepwise evaporated on each of the four substrates ranging in thickness from 0.2 nm to 200 nm (see experimental section for details on the deposition procedure). After each deposition step, the sample was heated in vacuum to 70 °C to remove excessive material and complete the perovskite conversion before it was transferred into the measurement chamber without breaking the vacuum.

**Figure 1:**
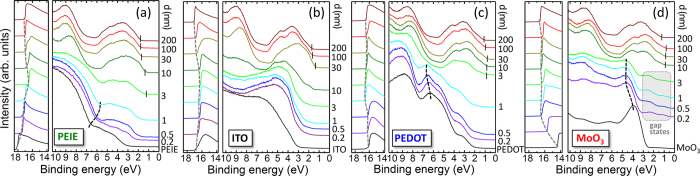
Overview over the UPS data taken on the four different substrate PEIE (**a**), ITO (**b**), PEDOT:PSS (**c**), and MoO_3_ (**d**); onto these, the perovskite MAPbI_3_ was incrementally evaporated with *d* being the deposited (intended) layer thickness. The left-hand panels show the changes in sample work function which are marked by grey dashed lines. The right-hand panels show the occupied DOS; here, the VB onset is marked by black vertical lines. The black dashed lines indicate changes in substrate specific features, showing that band bending is taking place. In (**d**) the appearance of gap states is marked by a gray area.

An overview over the UPS measurements of the perovskite films is shown in Fig. 1. In each panel, the substrate spectrum is given as lowest curve (black) while the following curves show measurements of the perovskite MAPbI_3_ at increasing layer thicknesses. From these measurements we can extract the changes in Wf with perovskite layer thickness (marked by grey dashed line) as well as the evolution of the valence band onset E_VB_ which are extracted from the spectra by linear extrapolation of the DOS (positions marked by vertical lines). For the substrates we find Wf(PEIE) = 3.11 eV, Wf(ITO) = 4.52 eV, Wf(PEDOT:PSS) = 5.12 eV, and Wf(MoO_3_) = 6.83 eV; these values are in good agreement to previously published work^35^,^36^,^37^. With increasing perovskite deposition, we observe a change in Wf for all samples, the direction of which depends on the substrate, thus, interface dipoles and band bending occur.

Looking at the valence band onset, it is quite notable that the layer thickness, after which the VB onset of the perovskite can be identified, as well as the thickness at which the typical shape of the perovskite DOS emerges, differs significantly between the substrate materials. On PEIE and PEDOT:PSS, perovskite formation starts more or less immediately (Fig. 1a and c), the typical shape appears after ca. 3 nm of precursor deposition. By contrast, it takes ca. 10 nm MoO_3_ (Fig. 1d) and even ≤30 nm on ITO (Fig. 1b) before perovskite formation is observed, making it impossible to identify changes in energy level alignment. The observation of such an induction period could be indicative of differences in surface coverage, i.e. island like growth; however, as will be shown below, it is rather an issue with the formation of the perovskite layer on the substrate surface. In addition, for MoO_3_, gap states appear at low coverage which have been reported previously for the inverse interface, when MoO_3_ was evaporated on top of the perovskite MAPbIBr_2_^24^.

Due to the considerable differences found in the formation of the perovskite films on the different substrates, it is necessary to look more closely at the film formation in the early induction period before further discussing the UPS data. For this, we can take advantage of the unique ability of XPS to identify not only the chemical elements, but also their redox state (i.e. chemical shift, bonding environment) as well as their relative composition (i.e. the film stoichiometry).

**Figure 2:**
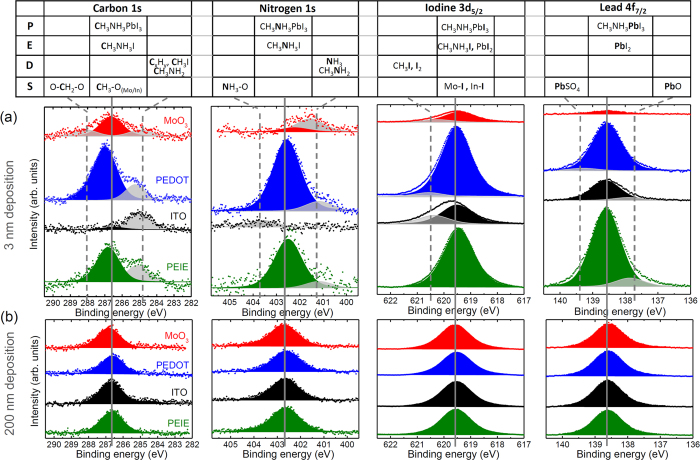
XPS carbon, nitrogen, iodine, and lead spectra of MAPbI_3_ evaporated on the 4 different substrates after (**a**) 3 nm and (**b**) 200 nm deposition. MoO_3_ is shown in red, PEDOT:PSS in blue, ITO in black, and PEIE in green. The solid vertical lines mark the expected binding energy in perovskite for each element, while the dashed lines show the positions of additional chemical environments observed at low coverage before the stoichiometrically correct perovskite is formed. The table above lists possible reaction products responsible for the additionally observed peaks, divided into products (P), educts (E), decomposition products (D), and surface bonds (S). Note that in some cases the binding energies of the peaks have shifted to enhance comparability as described in the text.

Figure 2 shows the XPS data of the carbon (C1s), nitrogen (N1s), iodine (I3d_5/2_), and lead (Pb4f_7/2_) signals after 3 nm as well as 200 nm of PbI_2_ and MAI precursor co-deposition on all four substrates; the spectra for the thinner (0.5 and 1 nm) as well as intermediate layers (10 and 30 nm) can be found in the Supplementary Information, Fig. S4. In some cases the substrate signals have been subtracted, which are carbon in the case of PEDOT:PSS and PEIE and nitrogen in case of PEIE. These features can be well distinguished because of the large difference in binding energy (see Supplementary Fig. S2 for original spectra). To further improve comparability and facilitate the discussion, the spectra for each substrate and thickness were shifted such that the Pb 4f_7/2_ peak (in the formal +2 redox state) occurs at E_B_ = 138.6 eV. By employing this procedure, effects due to the variations in the film work function are eliminated. As a result, the peak positions for C 1 s are centered at 286.7 eV (redox state +1), for N 1S at 402.6 eV (redox state +1), and for I 3d_5/2_ at 619.5 eV (redox state −1), all of which are in good agreement with previously published values [see e.g. refs 28,31,38,39]. The colored peaks in Fig. 2 mark these binding energies of the perovskite.

In the case of the thick layers (>30 nm), the XPS spectra appear identical on all four substrates, i.e. show a single binding energy for each of the four elements involved in perovskite formation (C, N, Pb, and I) indicative of the expected (identical) perovskite growth (Fig. 2b and Supplementary Fig. S4). By contrast, during the initial phase of layer formation (“induction period”), more peaks in additional bonding states of the participating elements are observed (marked in grey; Figs 2a and S4); these indicates that chemical reactions are taking place.

In the following, we will discuss the XPS results found after 3 nm of precursor deposition as shown in Fig. 2a; first the chemical shifts observed for the four elements (C, N, Pb, and I) are examined, followed by a discussion of absolute values (deposited material) and the relative content (stoichiometric composition). We would like to point out, that XPS cannot distinguish the educts, MAI and PbI_2_ (referred to as “E”), from the product MAPbI_3_ (referred to as “P”) as the chemical shifts are very similar. Further, the discussion must remain somewhat speculative, since a more detailed investigation of the decomposition products (referred to as “D”) or surface reaction products (referred to as “S”) would require additional sophisticated measurement techniques, like *in-situ* Raman spectroscopy, which is beyond the scope of this work.

Firstly, the carbon peaks will be discussed, which are positioned at 286.7 eV in the in compounds H_3_C-N^+^H_3_ I (E) and H_3_**C**-N^+^H_3_ PbI_3_ (P). On all substrates, additional peaks at lower energy (centered at ca. 284.9 eV) are observed, originating from products containing carbon in a less oxidized redox state as compared to E and P. As shown in the table in Fig. 2, the binding energy indicates that these are likely decomposition products (D) of MAI that physisorb on the surface, such as methylamine H_3_C-NH_2_^40^, methyl iodide H_3_C-I^41^, or higher hydrocarbons featuring C-C bonds^42^. These by-products can reach significant levels of the overall carbon signals; especially on ITO they dominate, as both E and P are hardly visible. On MoO_3_, a peak at higher energy (centered at ca. 288 eV) is observed as well, originating from products containing carbon in a more oxidized redox state, probably a chemical bond to surface oxygen (S)^42^.

Next, the peak centered at 402.6 eV represents nitrogen in the educt H_3_C-N^+^H_3_I (E) or the product H_3_C-N^+^H_3_PbI_3_ (P). On all substrates, except for ITO, additional peaks at lower energy (centered at ca. 401.2 eV) are observed, indicative of products containing nitrogen is a less oxidized redox state as compared to E and P. On MoO_3_, this species dominates over the perovskite signal, which itself is rather weak. We believe these to be decomposition products (D) of MAI, e.g. methylamine H_3_C-NH_2_ or ammonia NH_3_^43^. Surprisingly, on ITO nitrogen is hardly observed at all. There is merely a weak peak at higher binding energy (centered at ca. 403.8 eV) of products containing nitrogen in a more oxidized redox state, possibly nitride formed with oxidic surface (S)^44^. This leads to the conclusion that no perovskite is present here after 3 nm of precursor deposition.

The peak centered at 619.5 eV represents iodide ions **I**(-1) in both, E and P form. On all substrates, except for PEIE, additional peaks at higher energy (centered at ca. 620.5 eV) are observed, originating from products containing iodine is a less reduced redox state as compared to E and P. In agreement with the findings above we believe this to be again decomposition products of MAI, e.g. methyliodide H_3_C-I^41^ or I_2_.

Finally, the peak centered at 138.6 eV represents Pb(+2) ions in in both, E and P. On ITO and PEIE, additional peaks centered at lower energy (ca. 137.7 eV) are observed, from products containing lead in a less oxidized redox state compared to E and P. A possible explanation could be PbO^45^,^46^. On PEDOT:PSS, a peak at higher energy (centered at ca. 139.4 eV) is observed, indicative of products containing lead in a more oxidized redox state, possibly due to the formation of PbSO_4_^45^, resulting from the reaction of Pb(+2) with sulfate which is always present in PEDOT:PSS. No metallic Pb(0) is observed in any of the samples or layer thicknesses, which would be positioned around 136.8 eV^27^,^47^,^48^.

**Figure 3:**
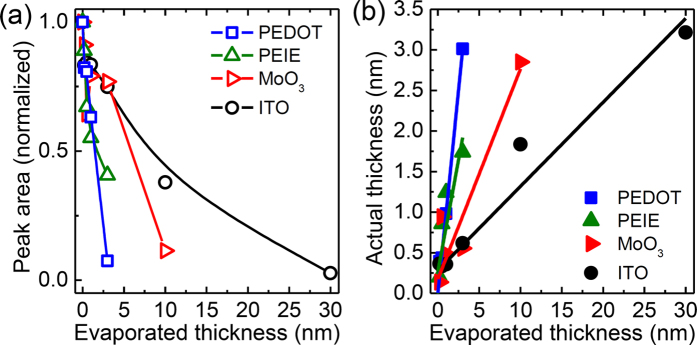
Determination of the actual layer thickness from XPS measurements: (**a**) Normalized peak intensities of S2p (PEDOT:PSS), Mo3d (MoO_3_), In3d (ITO), and N1s (PEIE) XPS core level signals depending on deposited (intended) thickness of the perovskite layer on top. (**b**) Actual layer thickness of the perovskite vs. deposited layer thickness as calculated from the attenuation of the core level peaks in (**a**).

Differences between the various substrates are not only found in the appearance of additional bonding states, but also the overall peak intensities vary considerably in Fig. 2a, indicating different layer thicknesses, even though nominally the same amount of precursor material has been deposited on all four substrates; in particular, all peaks appear lower on the two oxides, ITO and MoO_3_, compared to the organic substrates PEDOT:PSS and PEIE. In order to estimate the true layer thickness of the adsorbed material, the attenuation of substrate-specific peaks can be used, assuming an amorphous closed-layer growth. The XPS measurements for the S2p (for PEDOT:PSS), Mo3d (for MoO_3_), In3d (for ITO), and N1s (for PEIE) core level peaks are shown in Supplementary Fig. S1. By plotting the normalized peak areas as a function of the evaporated layer thickness d_evap_, as shown in Fig. 3a, it can be seen that on the organically-modified substrates, PEIE and PEDOT:PSS, the attenuation of the substrate peaks happens relatively fast (within deposition of ca. 3 nm of the precursors), while on the metal oxides, the substrate peaks can be observed much longer. Assuming a simple exponential dependency for the decrease in peak intensities with layer growth (Lambert-Beer law), we can estimate the actual layer thickness as shown in Fig. 3b. From these results we conclude that it takes approximately 10 nm of precursor deposition on MoO_3_ and even 20–30 nm on ITO for an effective 3 nm of material to stick to the surface.

The variations in peak intensity as well as the differences in substrate attenuation hint towards a surface-induced creation of volatile compounds, provoking the question, what happens to the precursor material that is missing on the substrates. Therefore, we propose several possible reaction channels for the formation of volatile compounds, as well as the decomposition products (D) described in the previous section. On the one hand, MAI might decompose into smaller fragments during the evaporation process. An indication for this is the rising background pressure when the deposition starts (from 10^−8^ to 10^−4^ mbar range) as well as the appearance of several fragments with mass smaller than 32 au in a mass spectrometer attached to the evaporation chamber. These fragments can either remain volatile, or physisorb onto the substrate, or react with each other and then deposit, giving rise to the additional peaks C, N, and I peaks in the XPS spectra observed at low deposited thicknesses (Fig. 2a). In the thicker perovskite films (Fig. 2b) these fragments are no longer observed in the XPS measurements, which indicates that they are not incorporated into the perovskite lattice, but rather leave the sample upon heating. We believe that this reaction channel is not very important, since otherwise the perovskite formation would be strongly hindered also later on.

More likely, the main reason for the vast differences observed between the substrates regarding film formation probably lie in the potential catalytic nature of the substrates. This is corroborated by the fact that the perovskite specific C and N peaks form readily on the catalytically inactive PEIE and PEDOT:PSS, while MAI is catalytically disintegrated on the oxide surface, leading to negligible amounts of C and N on ITO and MoO_3_ at low thicknesses. The reaction could be triggered by surface -OH groups which is a possible reason why ITO, which was stored in air before transfer to the UHV chamber, shows a much higher reactivity than MoO_3_, which was prepared *in-situ* and has thus a much lower initial density of hydroxyl surface groups. The high reactivity of ITO is, therefore, the reason why no nitrogen or carbon (+1) peaks are observed, i.e. most of the MAI decomposes during deposition of the first few nm of film formation.

The obvious lack in lead signal on the metal oxides in Fig. 2a, especially for MoO_3_, is the most surprising finding. The first option could be that the PbI_2_ does not stick to these substrates. However, this can be safely excluded, because when PbI_2_ is deposited on its own on the metal oxides, the actual and intended layer thickness coincide very well. A possible explanation is a surface-induced formation of tetramethyl-lead (Pb^+4^(CH_3_)_4_), which is known to be highly volatile. It has been previously reported that Pb^+4^(CH_3_)_4_, can be produced from inorganic Pb(+2) compounds and methyl iodide^49^. Here, the necessary oxidation of Pb(+2) to Pb(+4) would require redox activity of the substrate; experimental proof for this in the present study is only obtained from MoO_3_ which is indeed reduced from Mo(+6) to Mo(+5) (see Fig. S1). However, on ITO no similar change in either In or Sn oxidation states is found, so it is unclear how this oxidation of Pb takes place here.

Comparing the film stoichiometry after 3 nm of precursor co-deposition, as well as the observed oxidation states of the investigated peaks allows us to draw conclusions on the film composition at the interface; detailed films stoichiometries can be found in the Supplementary Information, Table S1. On PEDOT:PSS and PEIE approximately 30% of the layer is made up of perovskite at this point while the remaining material consists of physisorbed MAI, methylamine, and hydrocarbons. The intended and actual layer thickness coincide reasonably well, therefore the perovskite forms readily, even though mixed in with the byproducts. For evaporated layer thicknesses larger than 3 nm (Supplementary Fig. S4), no additional peaks are observed anymore and a pure perovskite is attained. This coincides very well with the point in the UPS spectra in Fig. 1 where the characteristic DOS emerges.

On ITO the effective layer thickness after three nm deposition is merely ~0.6 nm; no perovskite is formed due to the lack of nitrogen and we find mostly PbI_2_ as well as iodide bound to surface oxygen with additional traces of methyl iodide and hydrocarbons. In the case of MoO_3_, no perovskite is formed yet either; here it is due to the lack of Pb. Mostly iodide and carbon surface bonds are observed, as well as some MAI dissociation products. The effective layer thickness is approximately 1 nm.

Due to the formation of volatile compounds on the metal oxides, the film formation is hindered and the pure perovskite film can only start forming, once the metal oxide surface has been passivated by C and I surface bonds as well as MAI dissociation products; this passivation layer can be estimated to be approximately 3 nm thick and it takes ~10 nm and ≤ 30 nm of material deposition to form perovskite on MoO_3_ and ITO, respectively. Only at these points the additional XPS peaks vanish (Supplementary Fig. S4) and in UPS the perovskite DOS can be observed (Fig. 1).

**Figure 4:**
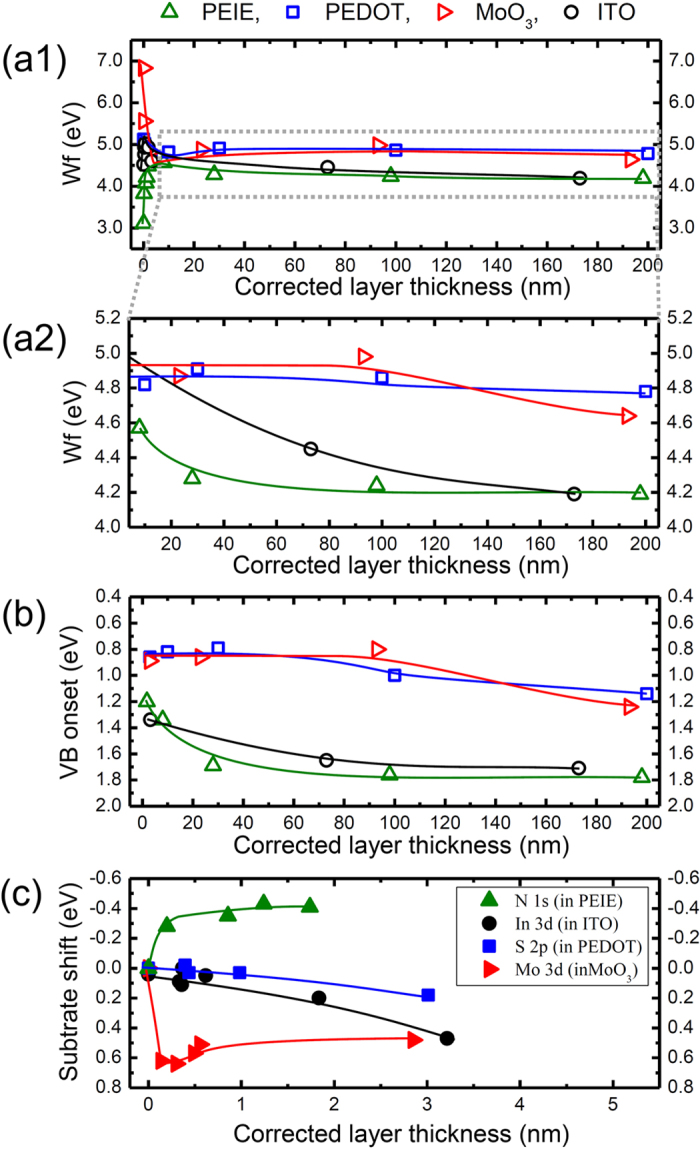
Changes in film properties of the MAPbI_3_ layer plotted vs. the corrected layer thickness: evolution of (**a**1) work function with (**a**2) showing only the Wf change at higher coverages, (**b**) valence band onset E_VB_, and (**c**) relative change in the binding energy E_B_ of substrate related peaks. Values of (**a1,2**) and (**b**) were extracted from the UPS-data in Fig. 1, while the XPS spectra used for (**c**) can be found in the Supplementary Fig. S1. Additional plots of the changes in core level positions can be found in the Supplementary Fig. S3.

Now that it is clear why the evolution of the valence band DOS differs between the UPS spectra of the four substrates in Fig. 1 we can continue the discussion of the UPS data and use the corrected layer thickness, taking into account the findings from Fig. 3b. For the first few nm of film formation, PEDOT:PSS and ITO only show minor changes in work function (ΔWf = 0.38 and 0.33 eV, respectively), while the more “extreme” substrates MoO_3_ (ΔWf = −2.19 eV) and PEIE (ΔWf = +1.08 eV) exhibit stronger shifts, mostly due to the formation of an interface dipole Δ as shown in Fig. 4(a1). Once the perovskite layer started forming, these initial shifts are followed by a gradual band bending with increasing thickness (Fig. 4(a2)). For the thick films, a Wf of approx. 4.7 eV is observed for the two substrates with higher work function (PEDOT:PSS: 4.78 eV and MoO_3_: 4.64 eV) while on ITO and PEIE a Wf of 4.19 eV is found in both cases. The fact that both these substrate materials lead to the same work function indicates that this is the lowest possible value, i.e. that the CB is pinned at the Fermi energy. These results are in good agreement with previously published findings from work function studies as mentioned in the introduction^23^,^26^,^30^,^31^,^32^.

For the valence band onsets E_VB_ plotted in Fig. 4b, a downward band bending is observed on all substrates, in agreement with the shift of the vacuum level; the XPS core level peaks show a similar shift in binding energy position, shown in the Supplementary Fig. S3. Once the passivation layer has formed this level bending takes approximately 100 nm for PEIE and ITO, while on MoO_3_ and PEDOT:PSS no saturation is observed even after 200 nm deposition. We can, therefore, not exclude that with further deposition of MAPbI_3_ the band bending would continue and, as a result, E_VB_ (and Wf) would further change. For the thick layers we find two different E_VB_, in agreement with the Wf measurements, a higher one for ITO (E_VB_ = 1.71 eV) and PEIE (E_VB_ = 1.78 eV), and a lower one for PEDOT:PSS (E_VB_ = 1.14 eV) and MoO_3_ (E_VB_ = 1.24 eV). From the combined Wf and E_VB_ measurements, the ionization energies can be calculated which are for the thick layers almost identical, yielding IE = 5.9 ± 0.1 eV, in agreement with previously reported values where the linear extrapolation of the density of states is used^50^,^51^.

To be able to draw energy level diagrams of these interfaces, the influence of perovskite coverage on the substrate energy levels has to be considered as well. Already in the UPS data, we can identify a shift of substrate associated features with increasing perovskite coverage (indicated by black dashed lines in Fig. 1). From the substrate specific XPS peaks already discussed in regard to the attenuation (Fig. 3 and Supplementary Fig. S1), we can extract the substrates’ specific binding energy shifts, as shown in Fig. 4c. We find significant changes in core level peak positions for all substrates due to charge transfer, especially for the samples with extreme Wf, i.e. MoO_3_ and PEIE. These indicate an electron transfer from the deposited layer to the substrate in case of MoO_3_, ITO and PEDOT:PSS, while in the case of PEIE the transfer is reversed.

**Figure 5:**
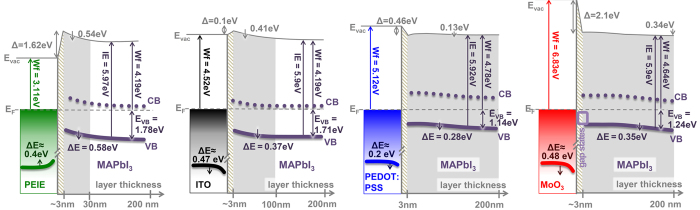
Energetic alignment at the interfaces of the four substrates as extracted from previously discussed UPS and XPS data. The schematics is divided in three regions: (i) interface region (shaded), (ii) band bending region (grey), and finally (iii) steady state alignment (white). The numbers stated at the substrate energy levels refer to the amount of band bending that is observed, the thickness indicated is corrected using the values extracted from Fig. 3b (E_VAC_ vacuum level, E_F_ = Fermi energy, E_VB_ = valance band onset, Δ = interface dipole, CB = conduction band).

The combined UPS and XPS measurements allow us to summarize the findings in energy level diagrams (Fig. 5), which can be used to estimate the performance of these interfaces in solar cells. Inside the perovskite layer we can distinguish three regions: (i) the interface region in which the passivation layer is formed and changes in vacuum level position are observed due to the modification of the substrate by surface reactions as well as charge transfer between the substrate and the overlayer (interface dipole); (ii) the band-bending region, where a gradual shift of the perovskite valence band is observed due to charge redistribution, and finally (iii) the steady state region with final band alignment. The downward band bending, which is present in all four cases, indicates that the prepared MAPbI_3_ is inherently n-type as often reported in literature^1^,^52^,^53^. However, the intrinsic charge-carrier density is rather low resulting in wide depletion regions, otherwise the same Fermi level position close to the CB would be reached on all substrates after the 200 nm of deposition.

Different demands are put on this interface, depending on the design of the device, e.g. solar cells. The perovskite can either be used as n- type semiconductor (type A devices: cathode/perovskite/hole conductor/anode) or as a p-type semiconductor (type B devices: anode/perovskite/electron conductor/cathode). In type-A devices, the interface to the underlying substrate determines electron injection/extraction. In our study, the substrates ITO and PEIE belong to this category. The relatively close proximity of the CB to the Fermi level indicates that these would be suitable as electron injection layers and enable a large open circuit voltage. However, the upward band bending towards the interface will hinder the extraction of electrons and could thereby reduce the solar cell fill factor.

For the high Wf substrates, which are candidates for type B solar cells, a shift of the Fermi energy towards the middle of the bandgap is observed, however, the hole injection barrier remains surprisingly high, around a value of ~0.8 eV and should therefore limit the achievable open circuit voltage. On the positive side, the observed band bending facilitates hole extraction.

The role of the ~3 nm passivation layer at the interfaces on the solar cell performance remains unclear at this point. In any case, it should hinder charge extraction from the perovskite to the bottom electrode. Further, the incorporated dissociation products could be mobile and migrate under voltage, leading to hysteresis as is commonly observed in type A devices^11^. In addition to being electronically relevant, the passivation layer probably also plays a role in film formation. Clear variations in film morphology are observed on the different substrates (see Supplementary Fig. S6), most probably due the different chemical nature of the passivation layer, which acts as the seeding and/or templating layer for further perovskite growth. It remains speculation, whether in solution-processed films the extent of byproduct formation is equally high as in the investigated thermally evaporated films, here. However, similar catalytic effects have been reported before in literature regarding the degradation of perovskite when prepared on ZnO substrates^54^,^55^; here, a decomposition of solution processed MAPbI_3_ films was observed under moderate heating and the degradation could be linked to the density of surface hydroxyl groups.

## Conclusion

In this study, we investigated the formation and energetic alignment of the hybrid perovskite MAPbI_3_ on the four different substrates PEIE, ITO, PEDOT:PSS, and MoO_3_. We observed the appearance of interface dipoles as well as band bending in the perovskite films. Therefore, the simplified assumptions of vacuum level alignment and flat band condition are not valid here. We find that the work function of the perovskite depends on the substrate, at least within the thickness range of 200 nm, and can be changed from 4.2 eV for the two low Wf substrates, such as PEIE and ITO, to 4.7 eV for the high Wf substrates, like PEDOT:PSS and MoO_3_.

More importantly, the substrate plays a crucial role for the film formation. Depending on the nature of the contact material, it takes between 3 and 30 nm for the density of states of the perovskite to emerge in the UPS measurement. XPS investigations show that this is due to chemical reactions taking place at the interface, especially in the case of the metal oxides whose catalytic action leads to the formation of volatile products. Only once the substrate is covered by physisorbed species creating a passivation layer, the perovskite film can start growing.

Our finding are in parts in contrast to the reports by Xu *et al*.^34^. As mentioned in the introduction, here a delayed formation of perovskite was reported as well, however, due to the formation of a pure PbI_2_ phase, with the conclusion that MAI does not stick to the interface. In our study clear signatures of PbI_2_ were only found on ITO, while on all surfaces carbon and nitrogen containing species dominated over the lead signal, indicating a sufficient sticking of MAI and to an even higher extend of MAI dissociation products. Clearly, more studies are needed to better understand the processes observed here. From the chemical shifts and film stoichiometries observed with XPS, plausible suggestions regarding the formed (volatile) degradation products can be made; more reliable tools, however, would be Raman or infrared spectroscopy which can identify the spectral signatures of the proposed products. Furthermore, the investigation of the typically used TiO_2_ interface will be of great interest; if similar byproducts are created here, they could explain the much stronger hysteresis observed in TiO_2_ based devices compared to PEDOT:PSS. The low-molecular-weight byproducts formed during deposition could easily migrate through the film under applied bias leading to changes in the IV-characteristics, while subsequently further reactions can set in at the interface.

## Methods

### Materials

Lead iodide was purchased from Alfa Aesar (ultradry, 99.999% metals basis) and used as received while MAI was synthesized according to literature^56^. The four substrates were prepared in the following ways: (i) ITO (Thin Film Devices Inc.) was cleaned ultrasonically (chloroform, aceton, deionized water) and used without further treatment. (ii) A PEDOT:PSS layer (Hereaus Clevios 4083) was spin cast at 2500 rpm from an aqueous solution onto ozone-treated ITO and annealed at 150 °C for 10 min resulting in a 40 nm thick layer. (iii) polyethylenimine ethoxylated (PEIE) was prepared on ITO using the method described by Zhou *et al*.^35^ leading to a film of 3–5 nm thickness. Lastly, (iv) 50 nm of MoO_3_ were evaporated *in situ* on top of an ITO sample.

### Perovskite film preparation

The incremental evaporation of the perovskite layers in deposited thickness steps of 0.2, 0.5, 1, 3, 10, 30, 100, and 200 nm was achieved by co-evaporation of MAI and PbI_2_ from two crucibles inside a vacuum chamber which is directly connected to the measurement system. The base pressure was 4·10^−8^ mbar, however during evaporation the pressure rose to ~4·10^−4^ mbar due to the volatile nature of the MAI molecule. For the deposition of each layer thickness, the evaporation rate of PbI_2_ and MAI were set to 0.4 Å/s and 0.6 Å/s, respectively and the deposition of the PbI_2_ was controlled by source as well as sample shutters; the rates were recorded by individual quartz crystal monitors (QCMs) that were previously calibrated, using 1.23 and 6.16 g/cm^3^ as densities for MAI and PbI_2_, respectively. However, it has to be noted that during the co-evaporation, the PbI_2_ QCM saw an additional rate of approximately 0.15 Å/s due to the volatile MAI that sticks to this quartz crystal as well and led to a (partial) transformation of the present PbI_2_ into perovskite. Therefore, the thickness read by the PbI_2_ QCM was assumed to be similar to the sample perovskite thickness. Scanning electron microscopy measurements (Supplementary Information Fig. S6) confirmed that these thicknesses are to a first approximation correct (error ~20%). After evaporation, the samples were heated in vacuum to 70 °C for 1 h to remove excess MAI and then transferred to the analysis chamber for characterization without breaking the vacuum. In prior experiments we found that this temperature of 70 °C is the highest possible in vacuum that does not lead to a degradation of the film to PbI_2_ during extended exposure. After the measurements were done, the samples were transferred back to the evaporation chamber for the next deposition step.

### Photoelectron spectroscopy measurements

These measurements were performed on a custom designed multi-chamber UHV system at a base pressure of 5∙10^−10^ mbar using a Phoibos 100 hemispherical analyzer (Specs) under normal emission to the sample. The electron binding energy scale E_B_ is calibrated using the Fermi edge of cleaned gold substrates. UPS measurements were conducted with a helium discharge lamp (He I @ 21.22 eV, sample bias −8 V) at a pass energy of 2 eV, with an energy resolution (as determined by the width of Fermi edge) of 110 meV. For XPS experiments, a Mg Kα excitation source was used (hν = 1252.6 eV) at a pass energy of 10 eV; the energy resolution is 800 meV. Despite of these energetic broadenings the uncertainties of the readout position (onset for UPS and peak positions for XPS) are rather precise and similarly prepared samples fabricated on different days usually agree within ± 50 meV. No degradation of the samples due to UV- or x-ray irradiation was observed.

### Peak Fitting

For the fitting of the XPS peaks, the parameters for full width half max (FWHM) and Lorentzian to Gaussian ratio (L:G) for each element were identified by looking at a wide variety of perovskite measurements and finding the best working fits. These parameters were kept identical for the specific element for all substrates and layer thicknesses. For the presented iodine, lead, and carbon peaks a Shirley background was subtracted, while for nitrogen a linear background worked best, due to the fact that the N1s peak is located on the slope of the Pb 4d_5/2_ peak. The relative sensitivity factors (RSF) needed for the determination of the perovskite stoichiometry were calibrated for the employed setup resulting in RSF(C1s) = 1, RSF(N1s) = 1.8, RSF(I3d_5/2_) = 32.8, and RSF(Pb4f_7/2_) = 16.5. The relative elemental concentrations were then calculated using the respective peak areas corrected by the RSF factors. From uncertainties in the RSF factors as well as the fitting procedure we estimate the relative error of the stoichiometry values to be in the range of 10%.

## Additional Information

**How to cite this article**: Olthof, S. and Meerholz, K. Substrate-dependent electronic structure and film formation of MAPbI_3_ perovskites. *Sci. Rep.* **7**, 40267; doi: 10.1038/srep40267 (2017).

**Publisher's note:** Springer Nature remains neutral with regard to jurisdictional claims in published maps and institutional affiliations.

## Supplementary Material

Supplementary Information
