# Supplementary material for: Substrate-dependent electronic structure and film formation of MAPbI3 perovskites
Source: Sci Rep. 2017 Jan 13;7:40267. doi: 10.1038/srep40267 (PMC5234022; doi:10.1038/srep40267)
Supplement: Supplementary Information [file srep40267-s1.pdf]

SUPPLEMENTARY INFORMATION

Substrate-dependent electronic structure and film formation of $MAPbI_3$ perovskites

Selina Olthof and Klaus Meerholz**

Department of Chemistry, University of Cologne, Luxemburger Straße 116, 50939 Cologne (Germany)

Dr. S. Olthof, Prof. K. Meerholz
Email: selina.olthof@uni-koeln, klaus.meerholz@uni-koeln

I) XPS measurements of the substrates' core level peaks

Substrate specific core level peaks have been measured using X-ray photoelectron spectroscopy (XPS) during increased deposition of the perovskite layer to investigate the effect of perovskite coverage on the energy levels of the substrates. Figure S1 shows the evolution of the N1s (PEIE), $In3d_{3/2}$ (ITO), S2p (PEDOT:PSS), and Mo3d (MoO_3) core level peaks. In the case of PEIE, the nitrogen signal originating from the perovskite layer is showing in the spectra as well, however as the binding energies of these nitrogen signals differ by approximately 2.4 eV, they can easily be separated in the data fitting process.

The red dashed lines in Fig. S1 show the shift of the substrate peaks. The corresponding values are shown as relative shifts in Fig. 4c in the main article. It has to be noted that in the case of MoO_3, not only a shift of the Mo3d peaks towards higher binding energy is observed, but a considerable broadening of the peaks takes place due to the emergence of a 5^+ oxidation state in addition to the expected 6^+ state of Mo in MoO_3. Approximately 80% of the surface layer is reduced due to the deposition of $MAPbI_3$. The peak shift plotted in the main article, Fig. 4c, corresponds to the shift of the 6^+ component.

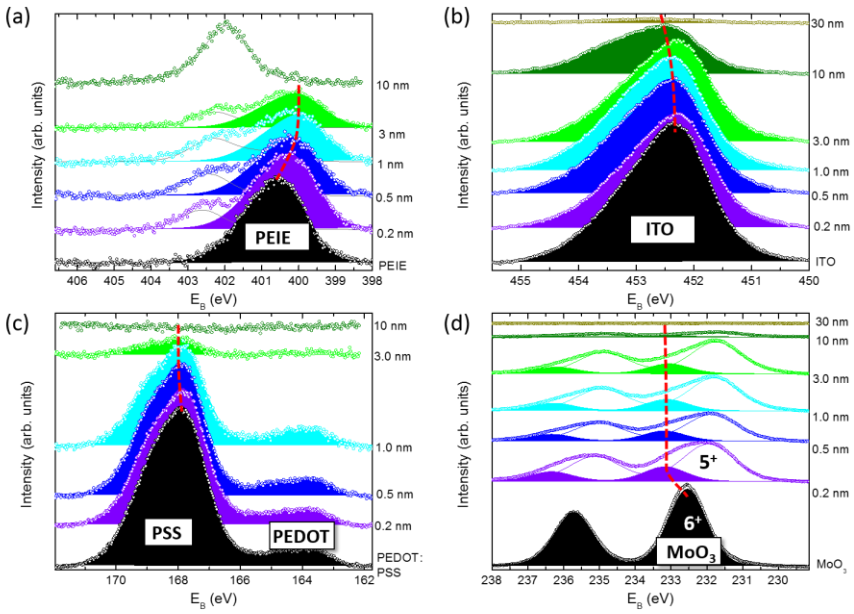

Figure S1: XPS spectra of the substrate specific peaks of the four substrates during the increased deposition of the $MAPbI_3$ film on top. Panel (a) shows the nitrogen 1s signal originating from the PEIE, (b) the In $3d_{3/2}$ peak of ITO, (c) the S 2p peaks from PEDOT:PSS, and (d) the Mo 3d peaks of MoO_3.

II) XPS measurements of the perovskite specific peaks sorted by substrates

Figure S2 shows all XPS measurements sorted by substrate (line) as well as element (column). In contrast to the data shown further below (Fig. S3), in this representation of the data, the substrate specific features have not been subtracted and are indicated by black lines in the peak fit.

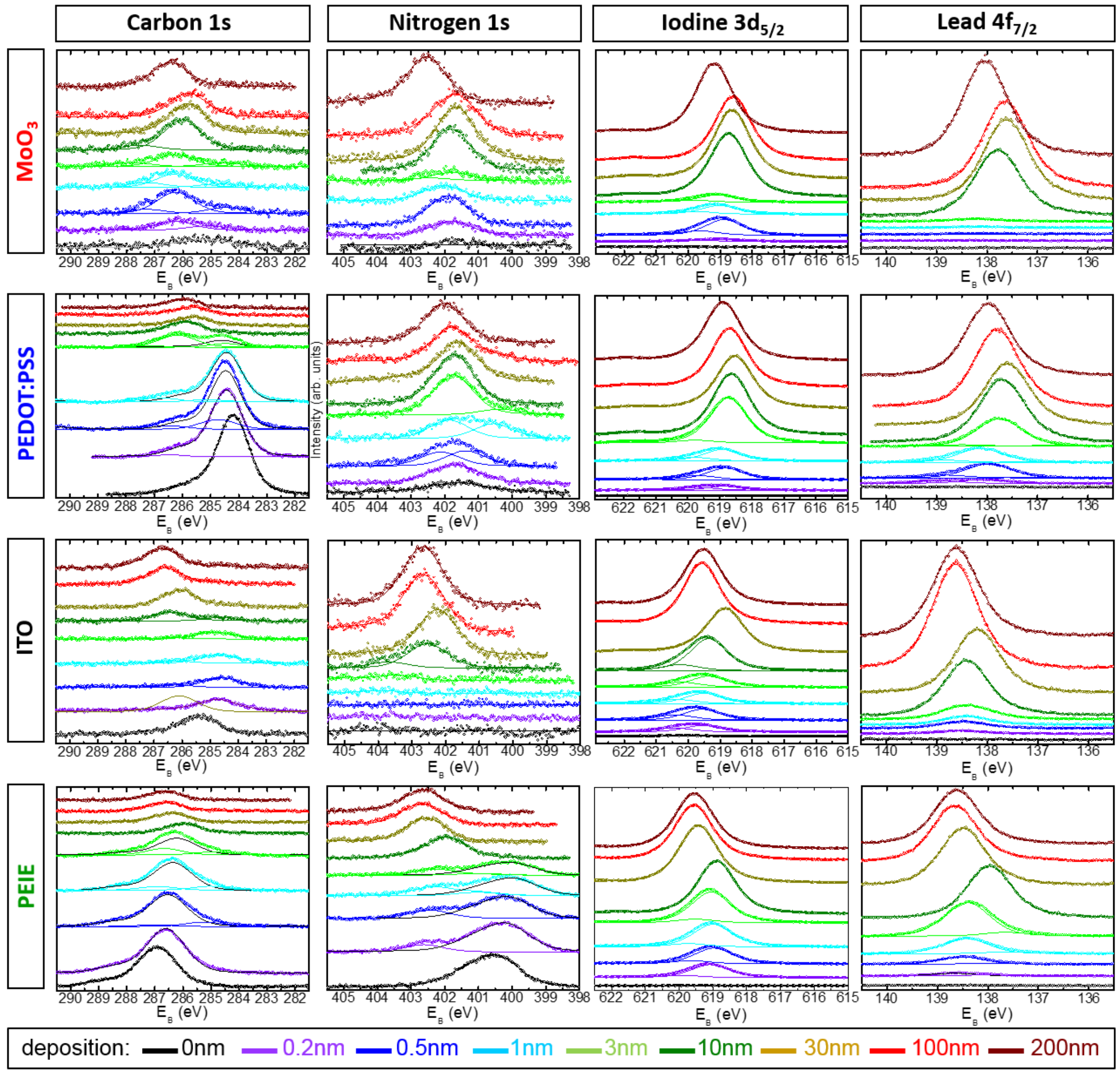

Figure S2. Original XPS measurements (with background subtracted) sorted by substrates (lines) as well as elements (columns) of the four samples MoO_3, PEDOT:PSS, ITO, and PEIE. Each panel shows the measurements for the incrementally evaporated $MAPbI_3$ ranging in intended layer thickness from 0 to 200 nm. The peaks were fitted by mixed Lorentzian and Gaussian curves, whereby the peaks shown in black originate from the substrate and the colored ones from the deposited perovskite.

The changes in XPS core level peak positions in Fig. S2 can be extracted in order to look at their thickness dependent changes. Thereby, band bending effects can be extracted, similar to the data obtained for the VB onset of the UPS measurement, shown in Fig. 4 of the main article. The changes in binding energy for the Pb4f, I3d and N1s peak are presented in Fig. S3. Here, the final value after 200 nm deposition is chosen as reference point (0 eV shift) and the

corresponding values of the VB onset shifts are included. As can be seen, once the perovskite starts forming after the passivation has been created, these shifts agree very well, showing therefore that core levels and the valence band density of state exhibit the same changes in energetic position, indicative of band bending.

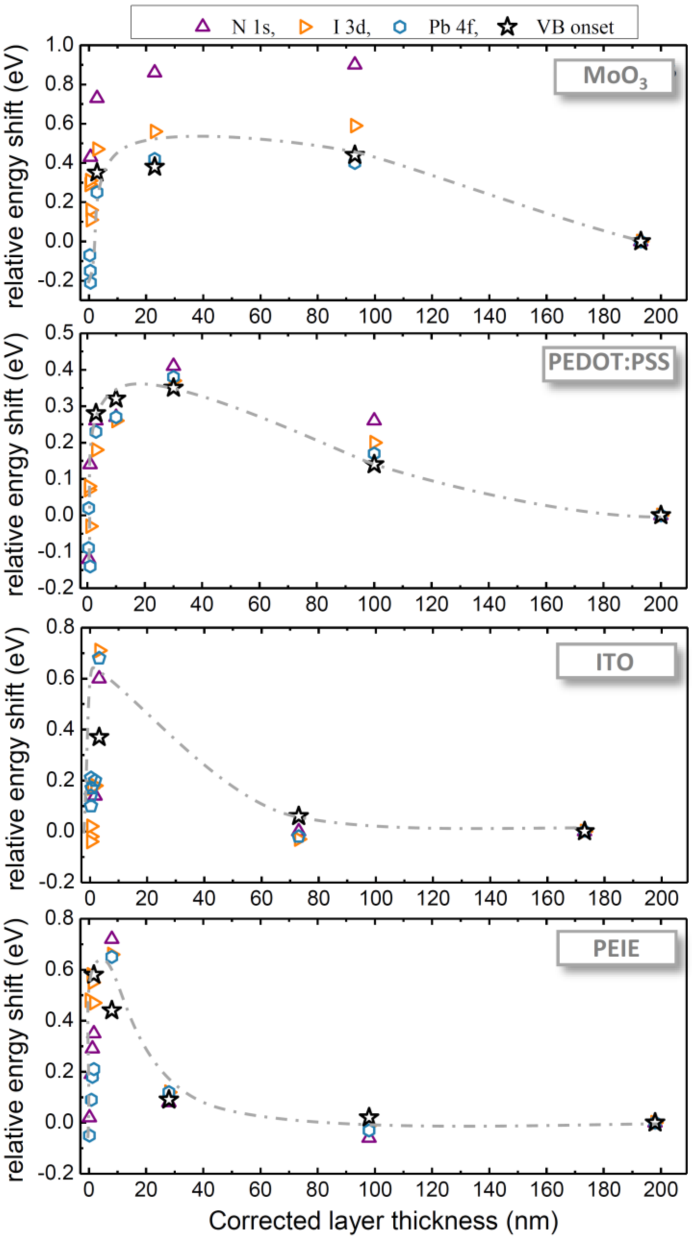

Figure S3: relative changes in the core level positions of N, Pb, and I which are furthermore compared to the changes observed in the VB as extracted from the UPS measurements. The thickness of the x-axis is corrected to show the actual layer thickness.

Figure S4. (next page) Same XPS measurements as in Fig. S2, this time showing a reduced dataset for comparison between the substrates; here, each line represents one layer thickness with the four substrates shown in comparison while the columns show the different elements C, N, I, and Pb. The solid colored peaks are at the expected binding energy for perovskite ("P"), while grey peaks indicate additional oxidation states which originate from unreacted educts ("E"), dissociation products ("D"), or surface bonds ("S"). The table above lists the possible reaction products. Note that the actual layer thicknesses differ from the intended ones and that in contrast to Fig. S2 the spectra have been shifted and substrate peaks are subtracted, all of which is described in more detail in the main article.

III) XPS measurements of the perovskite specific peaks sorted by deposited layer thickness

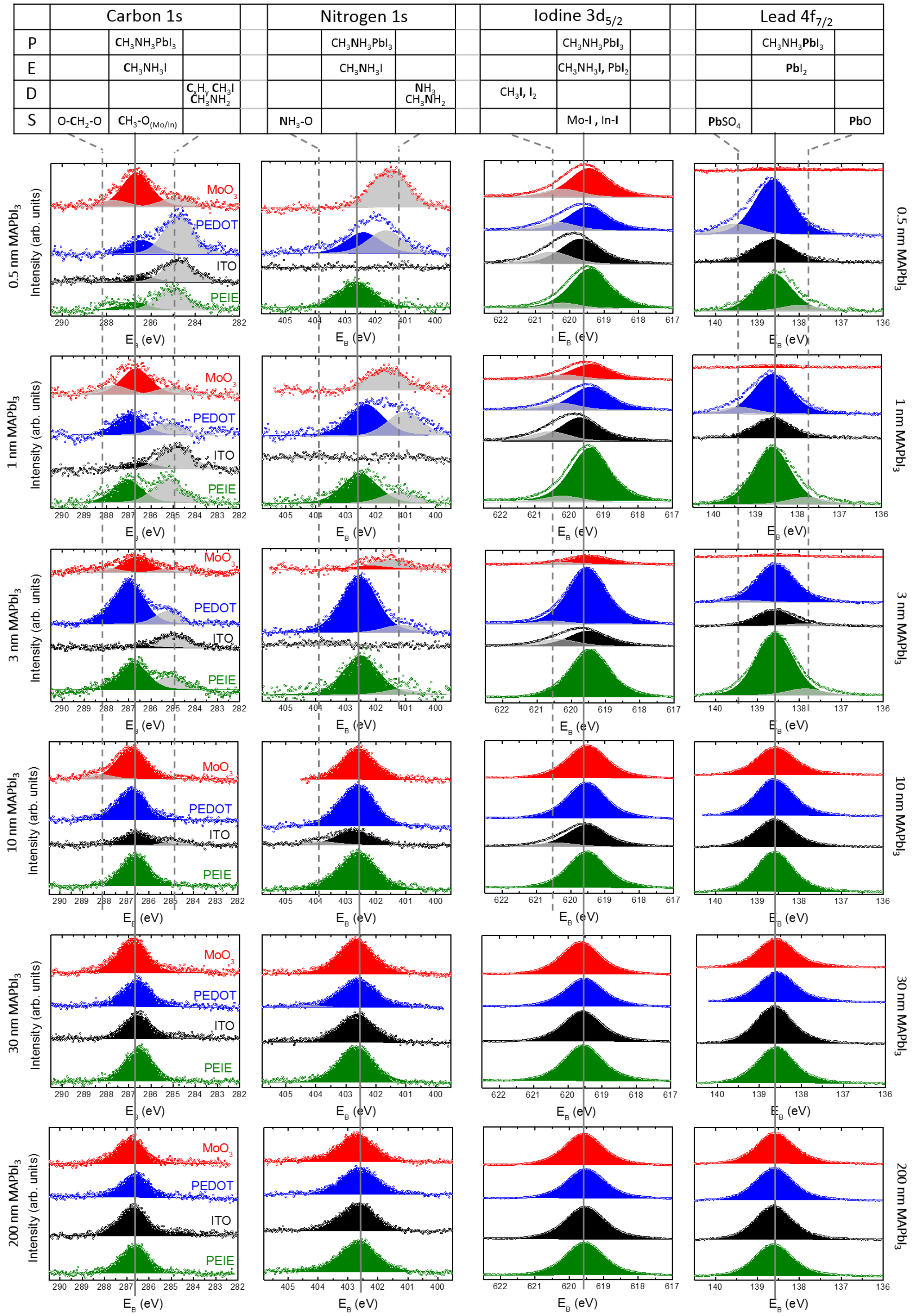

IV) Chemical reactions

In addition to the discussion of the XPS spectra in the main article, we would like to extend the analysis here to the full data set, covering deposited layer thicknesses from 0.5 nm to 200 nm as shown in Fig. S4. As described in more detail in the main article, the substrate related peaks have been subtracted and the spectra where shifted such that the Pb $4f_{7/2}$ peak is located at 138.6 eV. For the original spectra the reader is referred to Fig. S2.

In Figure S4, each line present the data from a specific layer thickness with the different substrates shown in red (MoO_3), blue (PEDOT:PSS), black (ITO), and green (PEIE), while the columns correspond to the elements. The solid colored peaks are at the expected binding energy for perovskite (indicated by P in the table on top of Fig. S4), while grey peaks indicate additional oxidation states which originate from unreacted educts (E), dissociation products (D), or surface bonds (S). Furthermore, the composition of each layer is listed in Table S1, where the iodide peak is used as reference to present the relative stoichiometry of the layers, taking into account both the perovskite as well as the by-product intensities.

PEDOT:PSS and PEIE

PEDOT:PSS and PEIE can be discussed together, as they show rather similar trends with increasing precursor co-deposition. For the thin films (0.5 and 1 nm) the carbon signal is dominated by a decomposition product at lower binding energy (284.9 eV) compared to the perovskite related peak at E_B = 287.6 eV; this peak originates from hydrocarbon, methylamine, or methyl iodide. These methylamine and methyl iodide species can be observed in the nitrogen and iodide measurement as well, where the according additional peaks show at higher binding energy for iodide (E_B = 620.5 eV) and lower binding energy for nitrogen (E_B = 401.5 eV). Note that on PEIE the neutral nitrogen peak is missing, most likely due to the fact that here the N signal originating from the substrate was subtracted which did overlie with this species. Regarding the lead peak, additional states are seen, created by surface bonds, which are associated with PbO in the case of PEIE and $PbSO_4$ for PEDOT:PSS. Especially for PEDOT:PSS, it is notable that this peak is rather strong at low thicknesses and then reduces quickly.

When the layer thickness is increased to 3 nm (which is the measurement discussed in the main article) the additional peaks quickly decrease in intensity, which means that the dissociation products are mostly only present at the interface, intermixing with the perovskite film as sketched in Fig. S5. After 10 nm of precursor evaporation, no additional peaks are present in the XPS spectra anymore and it can be concluded that here a pure perovskite layer has started forming.

Molybdenum oxide (MoO_3)

For the MoO_3 sample, we find again for low thicknesses a carbon signal at lower binding energy as well as an iodide at higher E_B, indicating the dissociation of MAI. In stark contrast to the organic substrates, here the nitrogen is present exclusively in a more neutral form for the 0.5 and 1 nm thick layers, so no perovskite forms and no MAI is physisorbed due to a surface induced disintegration of the MAI with the subsequent physisorption of amine,

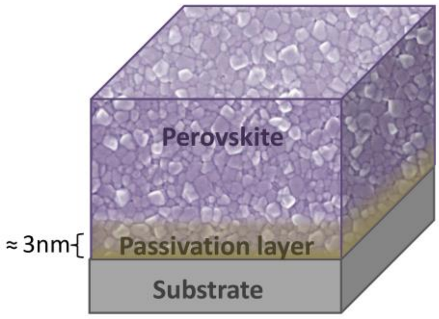

Figure S5. Schematic view of the formation of the perovskite film with a passivation layer forming at the substrate/perovskite interface.

methylamine, and methyl iodide species. Furthermore, almost no lead signal is detectable for deposition thicknesses below 10 nm. As discussed in the main article we postulate a surface-induced formation of volatile tetramethyl-lead ($(CH_3)_4Pb$). The fact that no lead and no nitrogen in the +1 oxidation state are observed, but still the iodide is mostly present as a more negative redox state, raises the question how iodine is bound, since it cannot be present as either PbI_2 or MAI. Therefore, we assume that the iodide undergoes a reaction with the substrate, possibly forming a MoO_2I surface layer. The excess carbon in the +1 oxidation state could be due to a further dissociation of the methylamine whereby the carbon forms bonds to surface oxygen atoms, breaking the double bond between Mo and O, forming O-CH_2-O. These two processes explain the observed change of the Mo oxidation state from +6 to +5 which is shown in Fig. S1d; approximately 80% of the molybdenum on the surface is hereby converted.

After the deposition of 10 nm of precursor material, the XPS peaks on MoO_3 mostly resemble the ones on the organic substrates, except for a remaining small carbon signal at higher binding energy, which finally is not observable any more after 30 nm of evaporation. Therefore, we can conclude that the pure perovskite layer starts forming between 10 and 30 nm deposition.

As discussed in the main article, the creation of volatile species reduceds the amount of actually adsorbed material, such that the intended and actual layer thickness do not coincide. The layer after 10 nm evaporation, when the perovskite starts forming on MoO_3, corresponds roughly to the point where effectively 3 nm of material have been adsorbed on the sample; the surface is therefore passivated by MAI dissociation products and the perovskite film is able to start growing.

Indium tin oxide (ITO)

Similar dramatic effects are observed for the ITO substrate. Here, only negligible amounts of nitrogen are found up to 10 nm deposition, therefore, we conclude that perovskite is not formed, and neither MAI nor other nitrogen-containing dissociation products are physisorbed to the substrate. On the other hand, most of the carbon is present in a more neutral bonding state (E_B= 284.9 eV), i.e. as adsorbed hydrocarbons and CH_3I (as further proven by the additional iodine peak). The main iodine peak at 619.5 eV could originate from perovskite, but as there is no corresponding N signal, it is must be present in the form of unreacted PbI_2, which has a comparable binding energy due to the same -1 redox state. However, there is more iodine present in the -1 oxidation state than can be explained by the PbI_2 formation, so similar to the case on MoO_3, probably surface iodide bonds form. Overall, the lead peak is much lower than for the organic substrates, so some amount of volatile lead compound (i.e.. tetramethyl-lead) must be formed as well, even though the effect is not as dramatic as on MoO_3.

In the case of ITO it takes even more precursor deposition than on MoO_3 before the corresponding peaks in Fig. S4 are comparable to the other substrates. After the evaporation of 10 nm, there are still significant additional oxidation states present and the overall peak intensities are much lower than on the other samples. Only after 30 nm of deposition, a pure perovskite phase is observed. So here the largest amount of volatile compounds is formed as confirmed by the substrate attenuation measurements in the main article (Fig. 3b) which show that it takes between 20 and 30 nm of deposition before the first 3 nm form on the surface; this will then act as a passivation layer to allow subsequent perovskite growth on this highly reactive surface.

V) Film stoichiometry

Based on the relative peak intensities and considering their individual measurement cross sections (RSF factors, see Experimental Section of main article), the XPS measurements of the C, N, I and Pb level peaks (Fig. S2/S4) allow to calculate the film stoichiometries, as shown in Table S1; as a reference point the iodide content is set equal to 3.

It can be seen that for thick layers the stoichiometry agrees rather well within the experimental error with the expected ratio of C:N:Pb:I equal to 1:1:1:3. By contrast, at low thicknesses a considerable excess of C, N, and I over lead is observed, in particular before the passivation layer is completed.

Table S1. Film stoichiometry calculated from the XPS peaks shown in Figure S2/S4. All intensities are given with respect to iodine (intensity set to 3). The colors mark the deviation from the expected film stoichiometry of C:N:Pb:I equal to 1:1:1:3 as indicated by the legend on the right. The given layer thickness corresponds to the intended thickness, not taking into account the volatile byproduct formation.

intended $MAPbI_3$ thickness	PEIE				ITO				PEDOT				MoO_3			
	C	N	Pb	I	C	N	Pb	I	C	N	Pb	I	C	N	Pb	I
0.5 nm	2.6	1.3	0.4	3	3.2	0.3	0.2	3	2.5	3.2	0.8	3	4.9	2.1	0.0	3
1 nm	2.5	1.3	0.5	3	2.6	0.2	0.3	3	3.4	3.4	0.8	3	6.4	2.2	0.1	3
3 nm	2.4	1.2	0.9	3	1.8	0.4	0.5	3	2.8	1.9	0.8	3	7.1	1.8	0.2	3
10 nm	1.5	1.3	0.8	3	1.1	0.8	0.8	3	1.5	1.3	0.8	3	1.9	1.0	0.6	3
30 nm	1.3	1.2	0.8	3	1.2	1.2	0.8	3	1.3	1.3	0.9	3	1.7	1.3	0.7	3
100 nm	1.3	1.2	0.9	3	1.1	1.1	1.0	3	1.1	1.0	1.0	3	1.3	1.2	0.8	3
200 nm	1.3	1.2	0.8	3	1.4	1.2	0.9	3	1.1	1.1	1.1	3	1.2	1.1	0.8	3

Legend: < 0.3; 0.3-0.5; 0.6-0.7; 0.8-0.9; 1; 1.1-1.2; 1.3-1.4; 1.5-1.7; > 1.7

VI) Film morphology

After the spectroscopic characterizations of the final layer thickness, the samples were removed from the UHV measurement chamber and transferred to a scanning electron microscope (SEM). For all four substrates, topography as well as cross section images were acquired, as shown in Fig. S6, in order to determine film quality and confirm the layer thickness. Variations in film morphology are observed on the different substrates, probably due the variations in chemical nature of the passivation layer that is formed differently on each of the substrate, which acts as the seeding and/or templating layer for further perovskite growth.

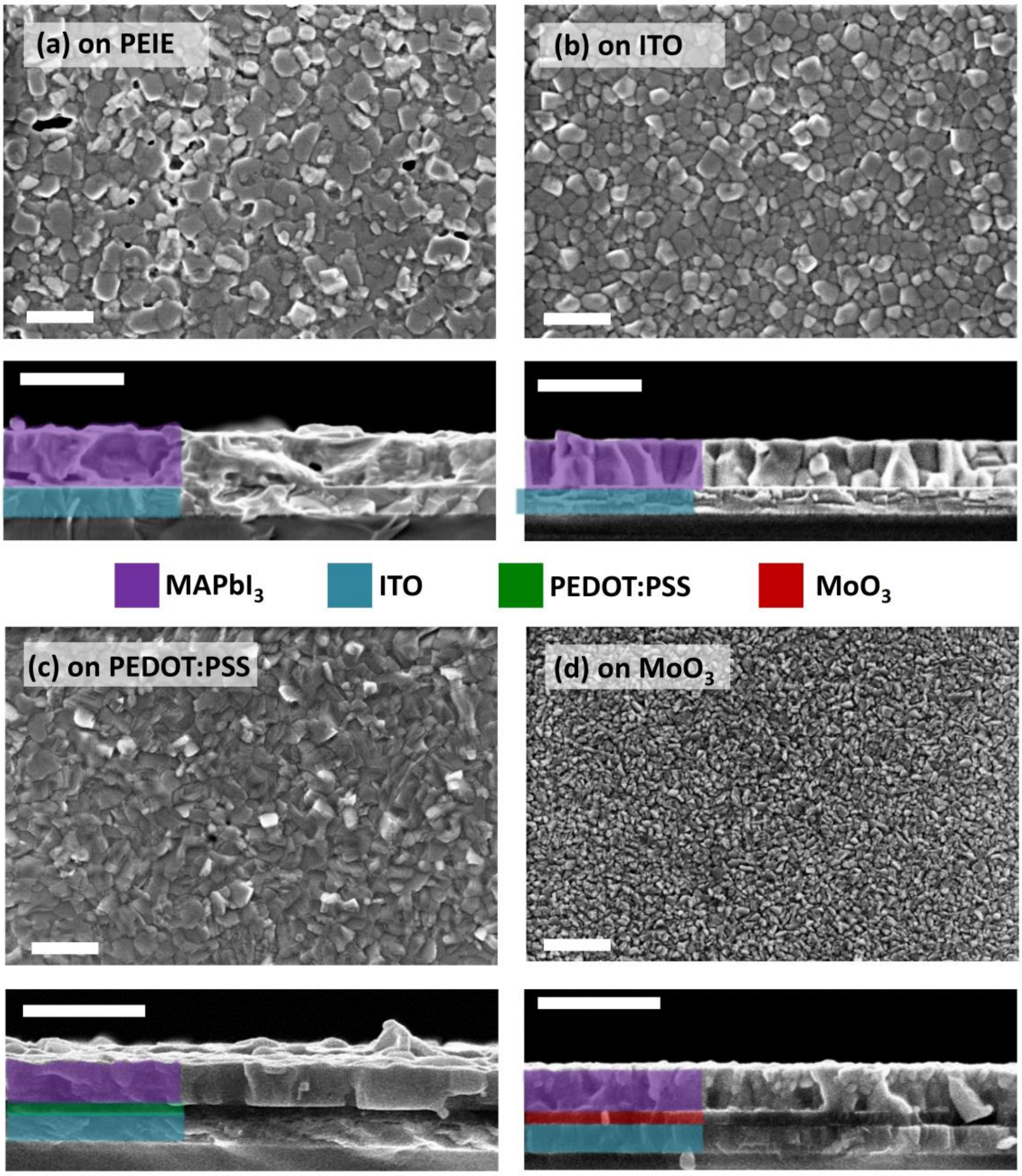

Figure S6. SEM measurements of the investigated $MAPbI_3$ samples, shown in top view as well as cross section. All scale bars are 500 µm long. For clarity, the different materials are partly highlighted by colors: Perovskite (purple), ITO (cyan), PEDOT:PSS (green), and MoO_3 (red).
